# Genome Survey Sequencing and Genetic Background Characterization of *Gracilariopsis lemaneiformis* (Rhodophyta) Based on Next-Generation Sequencing

**DOI:** 10.1371/journal.pone.0069909

**Published:** 2013-07-16

**Authors:** Wei Zhou, Yiyi Hu, Zhenghong Sui, Feng Fu, Jinguo Wang, Lianpeng Chang, Weihua Guo, Binbin Li

**Affiliations:** 1 Key Laboratory of Marine Genetics and Breeding, College of Marine Life Sciences, Ocean University of China, Qingdao, China; 2 Ocean School, Yantai University, Yantai, China; Li Ka Institute of Health Sciences, The Chinese University of Hong Kong, Hong Kong

## Abstract

*Gracilariopsis lemaneiformis* has a high economic value and is one of the most important aquaculture species in China. Despite it is economic importance, it has remained largely unstudied at the genomic level. In this study, we conducted a genome survey of *Gp. lemaneiformis* using next-generation sequencing (NGS) technologies. In total, 18.70 Gb of high-quality sequence data with an estimated genome size of 97 Mb were obtained by HiSeq 2000 sequencing for *Gp. lemaneiformis*. These reads were assembled into 160,390 contigs with a N50 length of 3.64 kb, which were further assembled into 125,685 scaffolds with a total length of 81.17 Mb. Genome analysis predicted 3490 genes and a GC% content of 48%.

The identified genes have an average transcript length of 1,429 bp, an average coding sequence size of 1,369 bp, 1.36 exons per gene, exon length of 1,008 bp, and intron length of 191 bp. From the initial assembled scaffold, transposable elements constituted 54.64% (44.35 Mb) of the genome, and 7737 simple sequence repeats (SSRs) were identified. Among these SSRs, the trinucleotide repeat type was the most abundant (up to 73.20% of total SSRs), followed by the di- (17.41%), tetra- (5.49%), hexa- (2.90%), and penta- (1.00%) nucleotide repeat type. These characteristics suggest that *Gp. lemaneiformis* is a model organism for genetic study. This is the first report of genome-wide characterization within this taxon.

## Introduction

*Gracilariopsis* was long indistinguishable from the genus *Gracilaria* in the family Gracilariaceae Nageli (Rhodophyta). In 1989, however, Fredericq and Hommersand [1] reinstated this genus based on four important differences between the two genera. Considered to be one of the most important economic macroalgae, *Gp. lemaneiformis* Bory is utilized for agar extraction [2], [3]. Its high agar yield accounts for more than half of total annual agar production worldwide [4]. *Gp. lemaneiformis* is also an ideal material for genetic research [5], and it has a potential role in the inhibition of red tides and in bioremediation [6], [7]. Single-rope floating raft cultivation of *Gp. lemaneiformis* has been widespread along the coastline of South China since the 1990s [5]. Currently, *Gp. lemaneiformis* is one of the most important aquaculture species in China [8].

Following the success of the human genome project, next-generation sequencing (NGS) technologies were launched. Compared with Roche's 454 and ABI's SOLiD, HiSeq 2000 offers the cheapest sequencing and the biggest output [9]. Illumina HiSeq 2000 also has been successfully used to characterize the genetic background of many marine species, including *Saccharina japonica* [10], *Pyropia yezoensis* [11], [12], *Membranipora grandicella* [13], *Anguilla japonica* [14], *Pinctada fucata* [15], *Pinctada martensii* [16] and *Pseudomonas stutzeri* AN10 [17].

Genetic studies of *Gp. lemaneiformis* aimed at improving its agar yield and growth rate are relatively new. Recent genetic studies have focused on the mitochondrial genome [18], functional gene cloning [19], analysis of genetic diversity [20], [21], and mutation research [22]. However, genetic studies of *Gp. lemaneiformis* remain underdeveloped compared with many other aquaculture species, such as *S. japonica*, *P. yezoensis*, *Cynoglossus semilaevis*, and *Apostichopus japonicus*, which might be due in part to the insufficient genetic or genomic resources available for *Gp. lemaneiformis*. To investigate and provide a genomic resource for further research (e.g., molecular cloning, structural and functional genomic studies, breeding, and comparative and evolutionary studies) on this species, we conducted a genome survey of *Gp. lemaneiformis* using NGS technology. The results of this study should be useful for crop improvement programs and better utilization of the existing genomic information in the future.

## Materials and Methods

### Sample preparation

A healthy female thallus of *Gp. lemaneiformis*, which was identified based on its morphology at maturity [23], was collected in November 2011 from the intertidal zone of Zhanshan Bay, Qingdao, China (36°02′ N, 120°20′ E). After clearing the specimen in seawater to remove epiphytes, mud, and sand, the thalli were rinsed with 1% sodium hypochlorite for 2 min and treated in antibiotic seawater containing 0.3 g L^−1^ penicillin, 0.2 g L^−1^ nystatin, 0.02 g L^−1^ cefotaxine, 0.1 g L^−1^ kanamycin, 1.0 g L^−1^ streptomycin sulphate, and 0.1 g ml^−1^ GeO_2_ for about 6 h to remove possible bacterial contaminants and diatoms. Part of a thallus then was used for DNA extraction, and the remaining thalli were cultured continuously in modified Provasoli's medium [24] under the following conditions: temperature of 20°C, photon flux density of 15 µmol m^−2^s^−1^, salinity of 30‰, and a 12 h light∶12 h dark photoperiod regime.

### DNA extraction, library construction, and sequencing

Genomic DNA was extracted using the Plant Genomic DNA Kit (Tiangenbiotech, Beijing, China) following the manufacturer's instructions. The quantity and quality of genomic DNA were measured using 1% agarose gel electrophoresis and Gene Quant (Amersham Bioscience, San Francisco, USA). Two paired-end libraries with insert sizes of about 170 base pairs (bp) and 500 bp were constructed from fragmented random genomic DNA following the manufacturer's instructions (Illumina, Guangzhou, China). Sequence data generation, using the Illumina HiSeq 2000 sequencing platform was conducted by Beijing Genomics Institute (Shenzhen, China). All sequencing reads were deposited in the Short Read Archive (SRA) database (http://www.ncbi.nlm.nih.gov/sra/), which are retrievable under the accession number SRX258772.

### Sequence assembly and analysis

The raw HiSeq 2000 reads were first pre-processed by trimming short tips and low quality sequences using a phred-scale quality score cut-off of 20 for the acquisition of Q20 values. Genome assembly was completed with the pre-processed reads using SOAPdenovo software [25]. All usable reads were realigned to the contig sequences, and then the paired-end relationship between reads was aligned between contigs. We constructed the scaffolds step by step using variant insert size paired-ends. Default assembly parameters of >40 bp overlap length and >90% sequence identity were used. The genome size was calculated using the total length of sequence reads and sequencing depth [26].

### Analysis of G+C content

We used 10-kb non-overlapping sliding windows along the assembled sequence to calculate GC content and average sequencing depth. The x-axis was GC content percent across every 10-kb window and the y-axis was average sequencing depth, which was determined across every 10-kb window independently.

### Identification of repeat sequences

Tandem repeats and interspersed repeats are two main types of repeats in the genome. The program TRF was used to search for tandem repeats. The annotated interspersed repeats were identified using RepeatMasker (version 3.3.0) and RepeatProteinMasker against the Repbase transposable element library (version 16.10). The software programs LTR-FINDER, Piler, and RepeatScout were used to construct a *de novo* repeat library, and then the software RepeatMasker was run to find homolog repeats in the *de novo* repeat library [27], [28].

### Gene prediction, annotation and comparison

Homology-based and de novo methods were used to predict genes, and the predicted results were integrated by the program GLEAN 17 [29]. Protein sequences from *Chondrus crispus*, *Cyanidioschyzon merolae*, *Pyropia yezoensis*, *Arabidopsis thaliana*, *Chlamydomonas reinhardtii*, *Chlorella variabilis*, and *Oryza sativa* were mapped to the genome assembly using blastN with an E-value of 1e-5 [30] to perform gene prediction, using one species each time. For de novo prediction, Augustus [31] and Genscan [32] were used to predict genes with parameters trained on *A. thaliana*.

Each predict gene was annotated by blastP to the GenBank database. Functional assignments were mapped onto Gene Ontology (GO) using Blast2GO and then the proportions of GO categories among four red algae species were compared using WEGO (http://wego.genomics.org.cn/cgi-bin/wego/index.pl).

### Synteny analysis of the genome

The two reference genomes (*C. variabilis* and *A. thaliana*) were downloaded from ftp://ftp.ensemblgenomes.org/pub/release-15/plants/fasta/arabidopsis_thaliana/(A. *thaliana*) and http://www.ncbi.nlm.nih.gov/genome/694 (*C. variabilis*), respectively. We used BLASTP [33] with an E-value threshold of 1e-5 to identify homologous genes between *Gp. lemaneiformis* and these two reference genomes. The program MCscan (version 8.0) then was used to identify syntenic blocks between the two genomes.

### Identification of simple sequence repeats (SSRs)

SSRs were mined in the genome sequence using the SSRIT program with the following parameters: at least six repeats for di- and four repeats for tri-, tetra-, penta-, and hexa-nucleotides for SSRs.

## Results and Discussion

### Sequencing and de novo short-read assembly

The two small-insert (170 and 500 bp) libraries were sequenced to generate a total of 21.52 Gb raw reads. After filtering and correction of the sequence data, a total of about 18.70 Gb of clean reads were obtained, with a read length of 95 bp and about 113.98X coverage of the estimated 160-Mb genome [34] (Table 1).

**Table 1:** Summary of two paired-end libraries used for HiSeq 2000 Sequencing and paired-end sequencing datasets.

Library	Sex of algae	Insert Size/bp	Read Length/bp	Data/Mb	Sequence Depth/X
L1	female	500	95	9,087.49	53.93
L2	female	170	95	9,608.67	60.05
Total				18,696.16	113.98

The program SOAPdenovo and the 18.69 Gb clean reads were used to conduct de novo assembly [25] to produce a contig with the N50 of ∼3.64 kb, longest contig length of ∼51.34 kb, and total length of ∼77.54 Mb (Table 2). A sequence with a scaffold N50 length of ∼20 kb, total length of 81.17 Mb, and longest scaffold length of ∼159,75 kb (Table 2) also was generated. Our draft genome assembly had unclosed gaps shorter than 16.3% (∼16 Mb) based on the calculation suggested in Li et al. [35]. In addition, Li et al. [35] reported that most gaps were easy to occur in repetitive regions with high unit identity and lengths larger than the sequencing read length, which usually could not be assembled with the current data.

**Table 2:** Statistics of the genome assembly.

	Contig		Scaffold	
	Size(bp)	Number	Size(bp)	Number
N90	127	94,867	127	57,716
N80	193	38,389	560	7,482
N70	648	15,203	5,754	2,422
N60	1,674	7,523	12,961	1,511
N50	3,638	4,379	20,007	1,013
Longest	51,340	-	159,753	-
Total size	77,537,041	-	81,167,384	-
Total number(≥100 bp)		160,390	-	125,685
Total number(≥2 kb)		6649	-	3,704

As sequencing depth increases, assembly quality also improves. Generally, current NGS assemblers require at least 30X coverage for a successful assembly without gaps from a standard multicell sample [36]. One of the most popular metrics to comparing assemblies is the N50 statistic [37], which is defined as a weighted median and is the smallest contig size in the set whose combined length totals 50% of the genome assembly [38], [39]. The N50 contig size of *Gp. lemaneiformis* (3.64 kb) in this study was larger than that of *Nannochloropis gaditanathose* (404 bp) [40] and *P. yezoensis* (1,669 bp) [11] but lower than those of prokaryotes with an average N50 contig size of 24 kb from de novo assemblies [41] or some terrestrial plants [26], [42], [43]. A large N50 contig and contig number might simply reflect a continuous and complete assembly [41]. However, it would be not valid to compare species using the contig N50 statistics from different assemblies if each N50 statistic was not calculated from the same combined length value [44].

### GC content and GC-depth analysis

**Figure 1:**
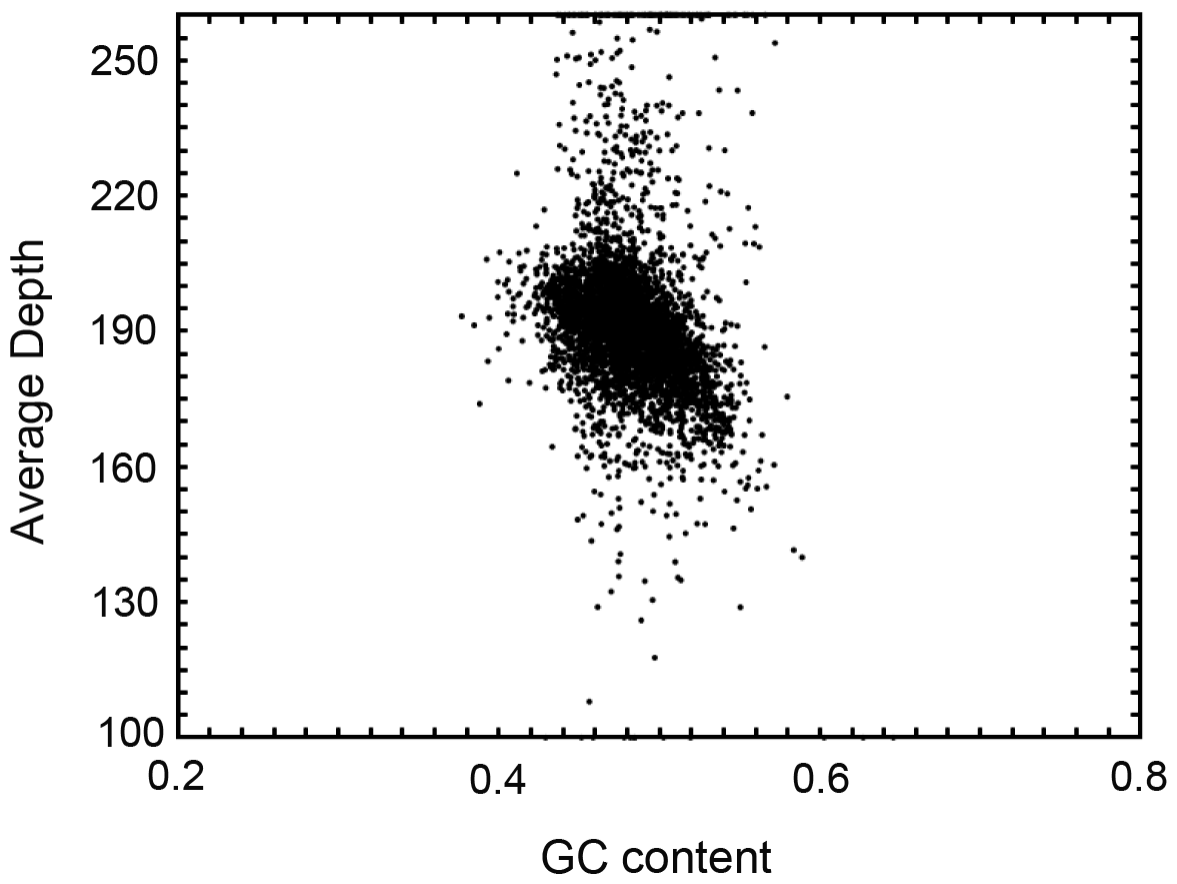
GC content and average sequencing depth of the genome data used for assembly. (The x-axis was GC content percent across every 10-kb non-overlapping sliding window).

Generally, genomic sequences generated through NGS are not uniformly distributed across the genome, as they are wider than the Poisson distribution [45]. GC content was one of three factors found to contribute to sequence bias from Illumina's platform [46]. Compared with mid-GC content, high and low GC contents cause reduced coverage in sequencing regions [47], [48]. To measure genome-wide sequencing bias, GC content and average sequencing depth were plotted using non-overlapping 10-kb sliding windows along the assembled sequence. The density points only concentrated in the 40–60% range, with the average GC content of ∼48%. Moreover, *Gp. lemaneiformis* had a mid-GC content [47], [48], which did not demonstrate abnormality of the species sample (some species themselves have abnormal GC content) or sequencing bias for the present data (Figure 1).

The 48% GC content in our study correlated well with values previously reported by Zhang et. al. [23], who found a G+C content of 41.1–49.7% in *Gp. lemaneiformis*. These values were higher than that of some marine bacteria (32.8–33.2%) including *Agarivorans gilvus*, *Aquimarina agarilytica* and *Vibrio tubiashii* [49]–[51] generated by Illumina paired-end sequencing; similar to values for marine macroalgae, such as *Solieria filiformis* (48.6%) [52], *Chondrus crispus* (46.3%) [53], *P. purpurea* (44.6%) [53], and *Laminaria hyperborea* (42.6%) [54]; and lower than that of *P. yezoensis* (63.6%) [11] and *Cyanidioschyzon merolae* (55.0%) [55].

### Estimation of genome size

**Figure 2:**
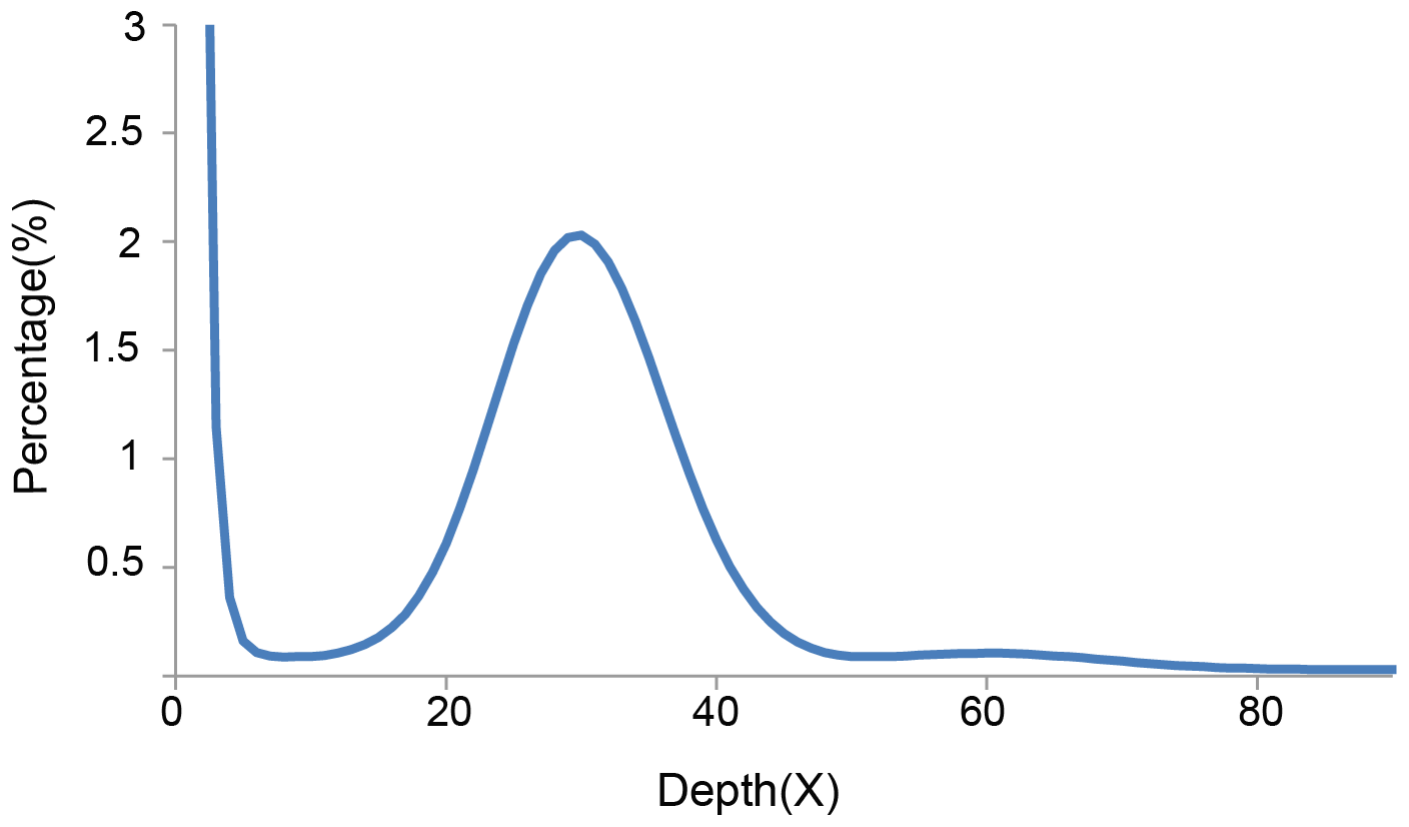
Distribution of 17-mer frequency in the 3.5 Gb sequences (3.5 Gb = 18.70 Gb*30X/162X).

Based on K-mer analysis, all sequences of about 18.70 Gb (Table 1) were used to estimate the genome size of *Gp. lemaneiformis*. The peak depth was at 162X, and we estimated the genome size to be 95.96 Mb. Generally, the 30X data are relatively accurate for estimating the size of the genome (based on evaluation experience of the Beijing Genomics Institute), so 3.5 Gb (18.70 Gb *30X/162X) of data were used to count and plot the distribution of 17-mer frequency. The peak depth was at 30X (Figure 2), and the number of 17-mers was 2,910,526,453 (Table 3). Their relationship could be expressed by the algorithm: Genome Size  =  K-mer_num/Peak_depth. The estimated genome size of 97.02 Mb (Table 3) was obtained, which was just one-quarter of the diploid size (375 Mb) for this alga based on average genetic substance content within the cells of *Gracilariopsis* [23]. The result also was not in accordance with the result of Kapraun and Dutcher [34], who reported a nuclear genome size of 160 Mb (haploid) in *Gp. lemaneiformis* using microspectrophotometry with the DNA-localizing fluorochrome hydroethidine. The same fluorescence method had been used to evaluate the genome size of *Ch. crispus* and obtained 150-Mb genome [53], which was about one-third larger than that of the complete genome sequencing method (105 Mb) [56]. We intend to verify the genome size using the flow cytometry method in the future. In total, haploid *Gp. lemaneiformis* appeared having a fairly small genome size, which would be an advantage for full sequencing of the genome.

**Table 3:** Estimation of *Gp. lemaneiformis* based on K-mer statistics.

K-mer value	K-mer number	Depth	Genome size (bp)	Used bases	Used reads	Depth (X)
17	2,910,526,453	30	97,017,548	3,500,000,165	36,842,107	36.07

Note: Generally, using 30X data to estimate the size of the genome are preference because of its accuracy (based on evaluation experience of the Beijing Genomics Institute); Used bases were calculated by 18.70 Gb*30X/162X; Genome Size  =  K-mer_num/Peak_depth.

*Arabidopsis* has been documented as a model organism for genetic study, mainly because it has a small genome (120 Mb genome) that is amenable to detailed molecular analysis [57], [58]. In marine macroalgae, *Pyropia* was suggested as a model organism for genetic studies, partly because its haploid has a relatively small genome size (diploid: 270–530 Mb) [12]. The estimated genome size of *Gp. lemaneiformis* (97.02 Mb) is similar to that of *A. thaliana* and *Ch. crispus* but much smaller than that of *Pyropia*, which shows the potential of *Gp. lemaneiformis* as a model species in this regard. Additionally, *Gracilariopsis* species reproduce both sexually and asexually and have a typical *Polysiphonia*-type life history that is completed in the laboratory within 6 months. These features make *Gp. lemaneiformis* a good model for studying genetics and genomics. It might also prove to be a model organism for common analyses of algal genetics and molecular biology.

### Repetitive sequences

Combined results from RepeatMasker and RepeatProteinMasker analyses revealed that transposable elements (TEs) constituted 54.64% (44.35 Mb) of the genome, and nearly one-third (20.46% of the genome) of them could not be classified within the TE regime (Table 4). Classification of the known TEs revealed that the majority of repeats were long-terminal repeat elements (LTRs) (26.40%), whereas 8.56% of the TEs were DNA transposons (Table 4).

**Table 4:** Percentage of the genome masked as each class of transposable elements.

Type	Repbase TEs		TE protiens		De novo		Combined TEs	
	Length (bp)	% in genome	Length (bp)	% in genome	Length (bp)	% in genome	Length (bp)	% in genome
DNA	228,529	0.28	2,138,557	2.63	6,682,133	8.23	6,950,958	8.56
LINE	63,656	0.08	117,208	0.14	1,210,955	1.49	1,342,261	1.65
LTR	2,255,161	2.78	7,687,608	9.47	20,671,304	25.47	21,425,837	26.40
SINE	3,153	0.004	0	0.00	0	0.00	3,153	0.004
Other	63	0.00	0	0.00	0	0.00	63	0.00
Unknown	0	0.00	0	0.00	16,608,826	20.46	16,608,826	20.46
Total	2,535,513	3.12	9,941,908	12.25	43,807,750	53.97	44,351,914	54.64

Note: RepBase TEs and TE proteins were obtained, using RepeatMasker and RepeatProteinMask respectively, based on the RepBase library; De novo repeat prediction identified repetitive DNA using RepeatMasker against the *de novo* repeat library of *Gp. lemaneiformis*, which was constructed by the programs LTR-FINDER, Piler and RepeatScout; Combined TEs were the integration and filtering redundancies of the above three methods.

The fraction of TEs in the *Gp. lemaneiformis* genome was similar to that of the pigeonpea (51.67%) [26]; higher than that of *Theobroma cacao* (24%) [59], rice (35%) [60], and animals such as *Cynoglossus semilaevi* (5.23%) [61], *Chlamys farreri* (15.84%) [62], and catfish (11.91%) [63]; and far lower than that of maize (85%) [64]. Repetitive sequences, especially TEs, are known to be prominent evolutionary factors that played a significant role in plant gene structure and genome evolution by enhancing genome plasticity, such as transposition, excision, insertion, chromosome breakage, and chromosome rearrangements [65]–[68].

In prokaryotes, DNA TEs are the major class of transposable DNAs, and RNA TEs are particularly abundant in eukaryotes [65]. Repeated sequences constituted 73% of the *Ch. crispus* genome, ∼55% (representing 58 Mb) of which were LTR retrotransposons [56]. As in *Ch. crispus* and some higher plants such as maize [69] and *Ricinus communis* [70], the most abundant retrotransposons in *Gp. lemaneiformis* are LTRs, which might be associated with potential horizontal transfer, insertional mutations, and heterochromatin near centromeres [71], [72].

### Gene prediction, annotation and comparison

Both homology-based and de novo gene prediction methods were used to predict the number of genes in the *Gp. lemaneiformis* genome (Table 5). First, Genscan and Augustus predicted 3369 and 3363 gene loci, respectively. Next, four homologous genome sequences were mapped to the genome assembly. Predicted genes 1,858–4,393 were obtained with an average transcript length of 787.06–910.23 bp. Subsequently, all data were integrated using GLEAN 17, and 3,490 genes were identified with an average transcript length of 1,429 bp, average coding sequence size of 1,369 bp, 1.36 exons per gene, exon length of 1,008 bp, and intron length of 191 bp.

**Table 5:** General statistics of gene prediction for *Gp. lemaneiformis*.

Method	Gene set	Number	Average transcript length (bp)	Average CDS length (bp)	Average exon per gene	Average exon length (bp)	Average intron length (bp)
De novo	Augustus	3369	631.62	558.01	1.35	413.99	211.59
	Genscan	3363	2330.66	1688.73	2.68	631.02	382.97
Homolog	*Cy. merolae*	1860	766.51	699.30	1.37	510.34	181.52
	*Ch. Crispus*	1858	773.80	702.59	1.36	515.61	196.36
	*P. yezoensis*	1878	764.10	696.32	1.37	509.31	184.59
	*A. thaliana*	4222	879.14	843.62	1.32	638.08	110.27
	*C.reinhardtii*	4039	808.82	746.46	1.27	586.79	229.19
	*O. sativa*	4393	910.23	802.41	1.34	597.55	314.52
	*C. variabilis*	3851	787.06	746.58	1.28	583.54	144.88
GLEAN		3490	1429.57	1369.79	1.36	1008.36	191.13

Note: Gene length included the exon and intron regions but excluded UTRs.

The number of predicted genes in the genome of *Gp. lemaneiformis* was comparable to that of the ultrasmall unicellular red alga *Cy. merolae*,(5331) [55] but the number was much lower than that of other sequenced genomes such as *Arthrospira platensis* (6153) [73], *Nannochloropis gaditana* (9052) [40], *Ch. crispus* (9606) [56], *Ascaris suum* (18,542) [74], and *Cucumis sativus* (26,682) [75]. Possible reasons for these differences include insufficient sequence depth coverage, variable regulation of gene expression levels (such as an enormous wealth of alternative splicing), and low sequence homology because of shorting of gene information from closely related species.

In comparison with other red algae (Table 6), *Gp. lemaneiformis* genome showed similar genome characteristics, such as average CDS length, short exon/intron length (bp) and low number of exons per gene. Especially, the genes of *Gp. lemaneiformis* also had few introns and nearly three quarters of predicted genes are monoexonic, resulting in a low average number of introns per gene (0.36). The high proportion of monoexonic genes for *Gp. lemaneiformis*, which was comparable to those of other red algae, further verified Collén et al.'s conclusion that a low number of introns were a typical characteristic in red algal genomes [56].

**Table 6:** Comparison of general genome characteristics from four red algae.

Species	*Gp. lemaneiformis*	*Ch. crispus*	*Cy. merolae*	*P. yezoensis*
Average CDS length (bp)	1370	-	1552	1247
Average exon length (bp)	1008	789	1540	755
Average intron length (bp)	191	123	248	300
Introns per gene	0.36	0.32	0.005	0.7
Exons per gene	1.36	1.32	1.005	1.7
Intron-containing genes (%)	28	12	0.6	∼40

**Figure 3:**
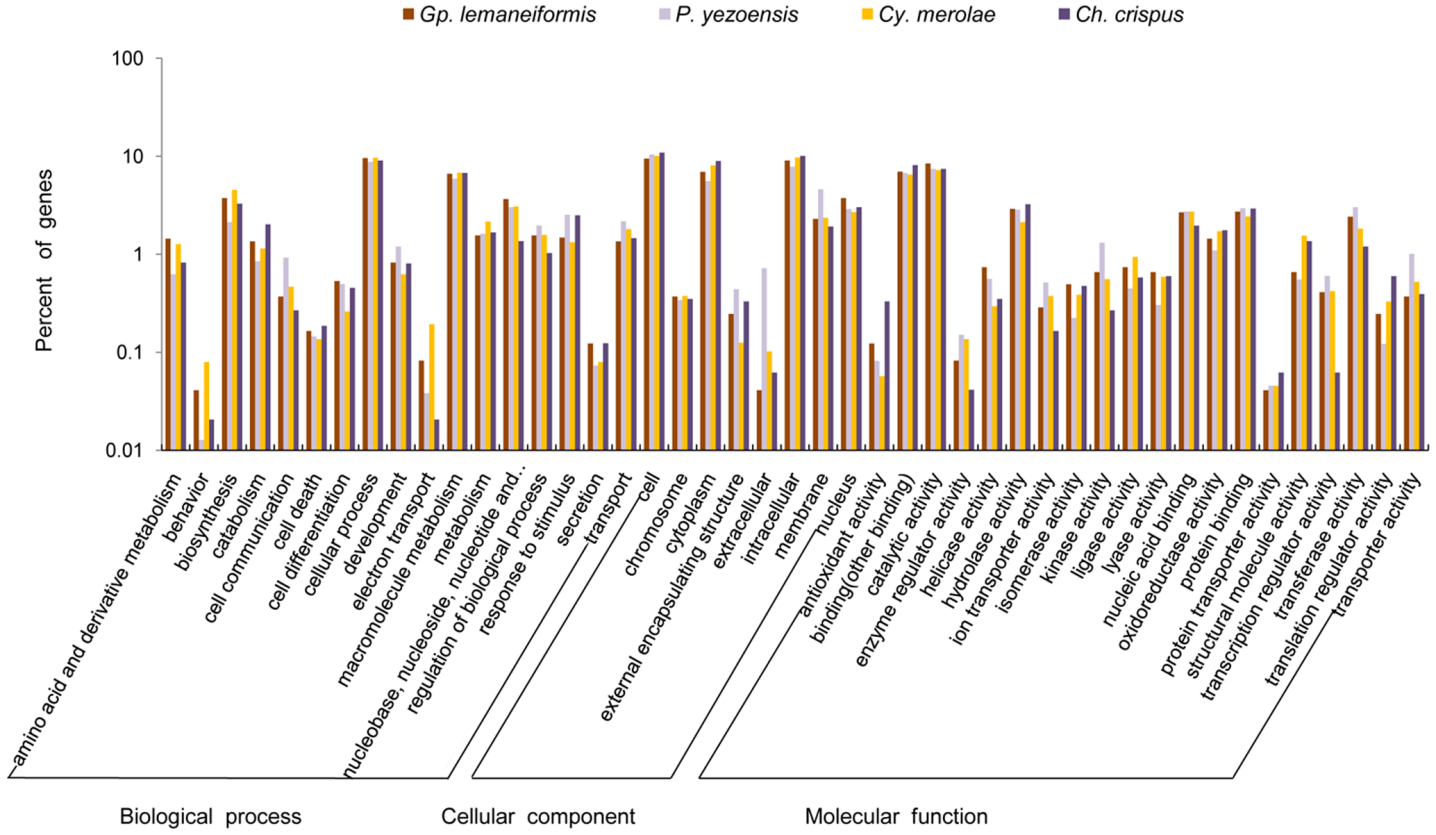
GO category comparison among *Gp. lemaneiformis*, *P. yezoensis*, *Cy. merolae* and *Ch. crispus*.

Of the 3,490 predicted genes in the *Gp. lemaneiformis* genome, 2,954 genes matched known genes in GenBank and 15.35% (536) were unknown. 2,430 genes could be assigned to one or more ontologies. Totally, 34.57% genes were grouped under biological processes 32.26% genes under cellular components, 33.17% genes under molecular functions. Furthermore, cellular process (27.86%) was the most highly represented groups under the biological process category, followed by macromolecule metabolism (19.17%); for the cellular component, intracellular (28.19%) and cytoplasm (21.56%) part were the significantly represented groups; a relatively high proportion of genes (25.43%) were involved in catalytic activity. The predicted proteins were compared with the 10,327 proteins from *P. yezoensis* [11], 9,606 proteins from *Ch. crispus* [56] and.5,331 proteins from *Cy. merolae* [55]. As shown in Figure 3, the vast proportion of GO categories did not show significant differences among 4 red algae except those related behavior process, electron transport process and extracellular component, which hinted that the biological processes of the above among 4 red algae were different.

### Synteny with sequenced plant genomes

We compared about 81.17 Mb of the genomic sequence from *Gp. lemaneiformis* with the *A. thaliana* and *C. variabilis* genomes using the program LASTZ, and then MCscan was used to identify syntenic blocks. The genomic sequence of *Gp. lemaneiformis* showed minimal matching to *A. thaliana* (match length of 900,618 bp with only 1.11% coverage) and *C. variabilis* (match length of 345,067 bp with only 0.43% coverage),and the minimal matching could not be used to evaluate the general features of synteny relationships (Tables 7 and 8). Although it should be possible to analyze the evolutionary history between two species using complete genomic sequences [76], the following factors would make the process difficult or impossible: short sequence length of initial assembly, low gene distribution, and species specificity.

**Table 7:** Summary of match sequence between *Gracilariopsis lemaneiformis* and *Arabidopsis thalianai*.

Species	Match length (bp)	Total length (bp)	Coverage (%)
*A. thaliana*	1,671,544	119,146,348	1.40
*Gp. lemaneiformis*	900,618	81,167,384	1.11

**Table 8:** Summary of match sequence between *Gracilariopsis lemaneiformis* and *Chlorella variabili*.

Species	Match length (bp)	Total length (bp)	Coverage (%)
*C. variabilis*	762,232	46,159,512	1.65
*Gp. lemaneiformis*	345,067	81,167,384	0.43

### SSR detection

**Figure 4:**
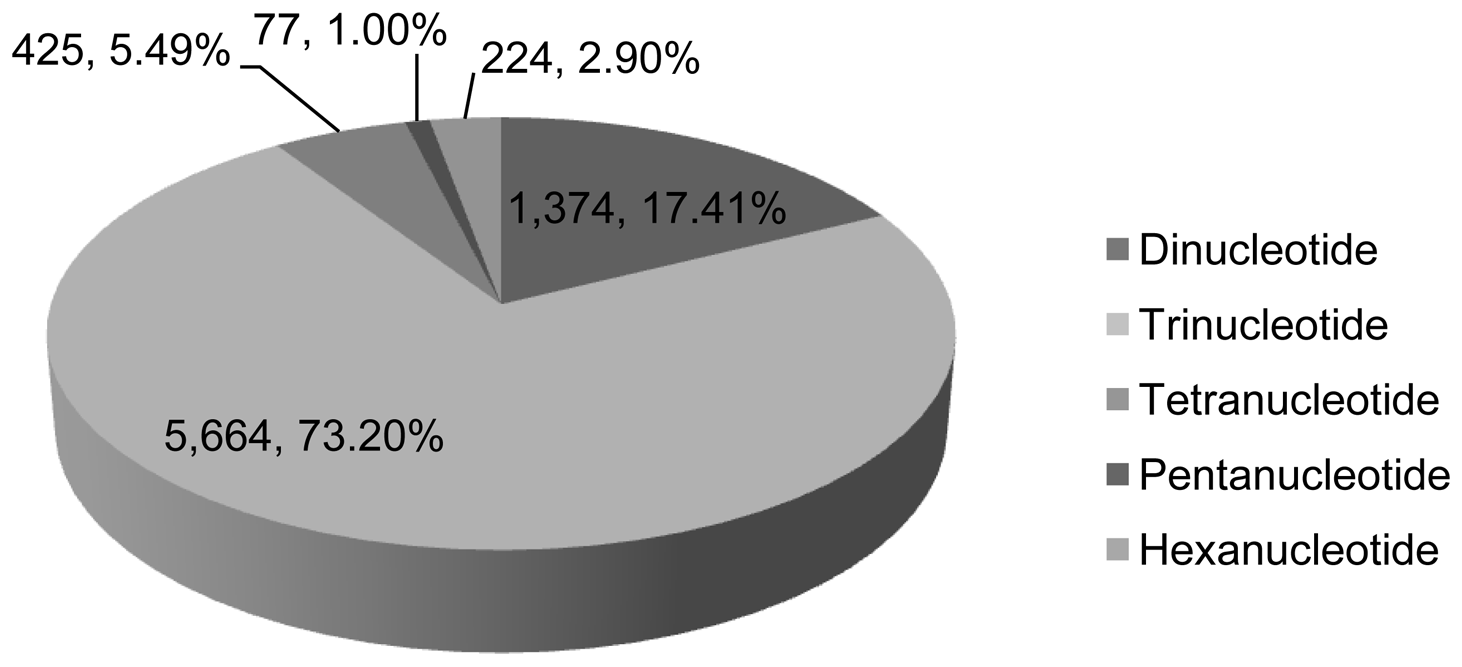
Frequency of SSR types in the Genome Survey of *Gp. lemaneiformis*.

In this study, we found 7737 derived SSRs, none of which were mononucleotide repeats and complex SSR type. Among these SSRs, the trinucleotide repeat type was the most abundant (up to 73.20% of total SSRs), followed by di- (17.41%), tetra- (5.49%), hexa- (2.90%), and penta- (1.00%) nucleotide repeat types (Figure 4).

**Figure 5:**
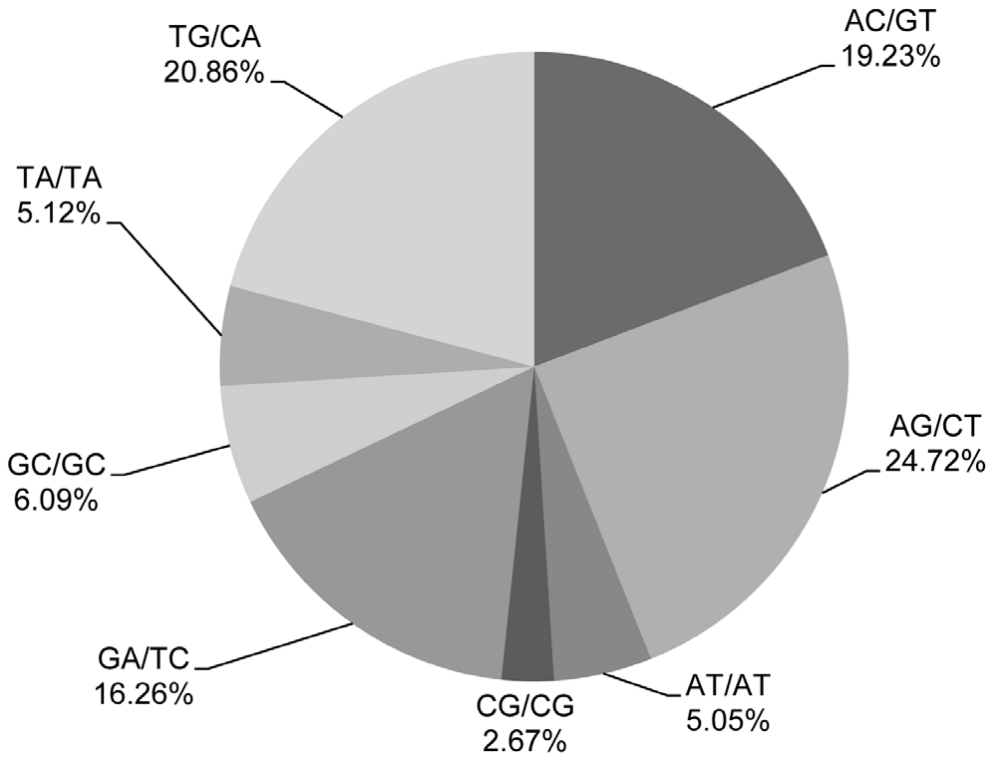
Percentage of different motifs in dinucleotide repeats in *Gp. lemaneiformis*.

**Figure 6:**
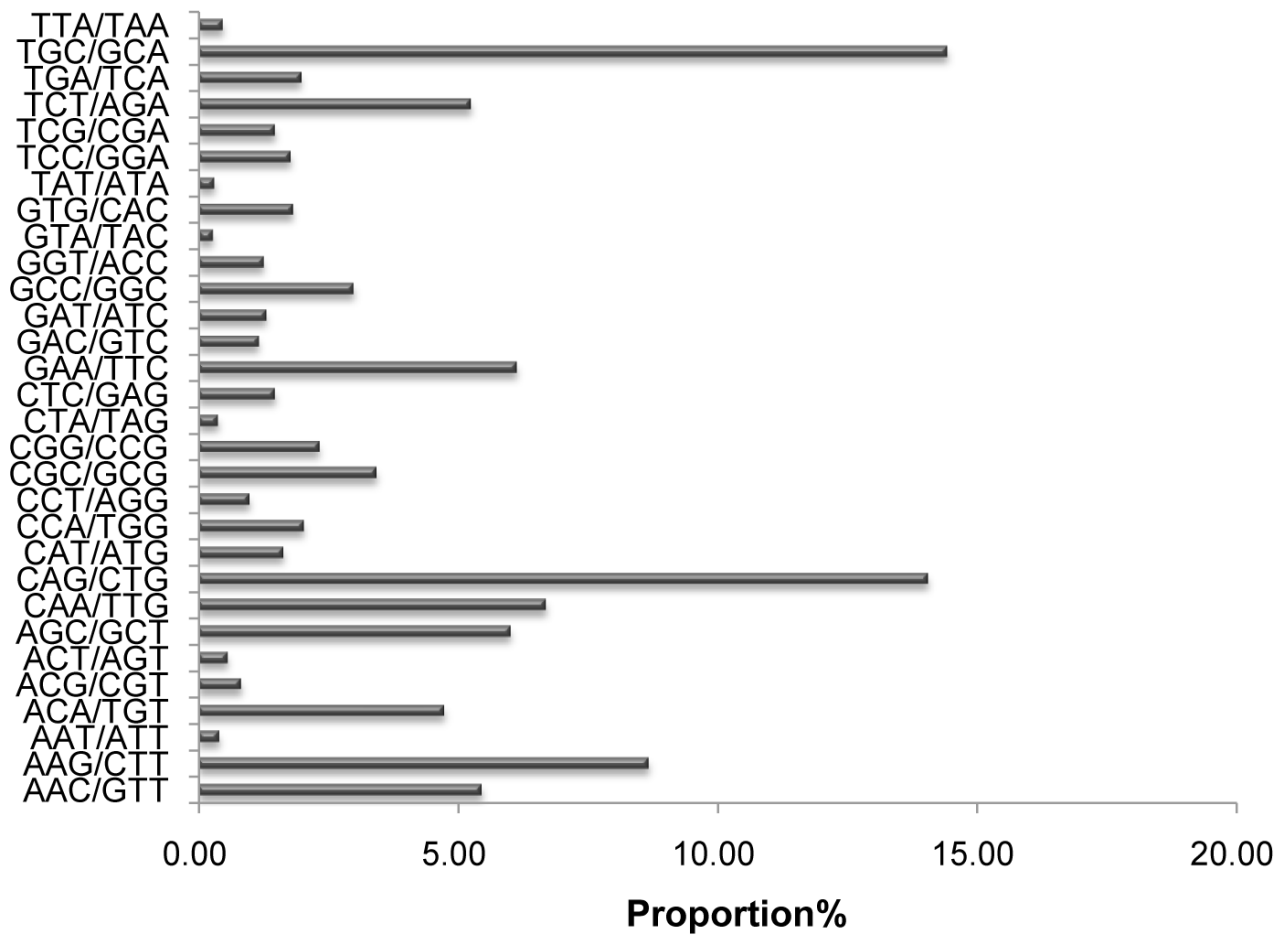
Percentage of different motifs in trinucleotide repeats in *Gp. lemaneiformis*.

In addition, 315 motif types were identified, which consisted of di- (8), tri- (30), tetra- (68), penta- (56), and hexa- (153) nucleotide types. Table S1 shows the SSR frequency of each motif. Statistical analysis revealed that the motif AG/CT was the most abundant among the dinucleotide repeat motifs, accounting for 24.72%, followed by TG/CA at 20.86% (Figure 5). The most prominent dinucleotide motif of *Gp. lemaneiformis* was the same as that of the rubber tree, in which the AG/CT motif was also most abundant [41]. In embryophytes, yeast, and fungi, however, the AT/AT motif is most abundant [77]. Within the trinucleotide repeat motifs, the common motifs TGC/GCA and CAG/CTG accounted for 14.42% and 14.05%, respectively, in *Gp. lemaneiformis* (Figure 6).

In the *Gp. lemaneiformis* genome, the trinucleotide repeat type was predominant, as is also the case in other species such as *P. yezoensis*, *A. thaliana*, rice, maize, and tomato [12]. Yang et. al. [12] reported that the trinucleotide repeat type in the coding regions would enhance gene variation but not cause frameshift mutation. SSRs are inherently unstable, which creates and maintains quantitative genetic variation, so they must have played an important role in genome evolution [77]. It is possible that the 7737 derived SSR loci found in our study may be used as SSR markers for genetic mapping in the short term.

## Supporting Information

Table S1 **Occurrence of SSR motifs in Genome Survey to** ***Gracilariopsis lemaneiformis*** **.**
(DOC)
